# 基于非靶向代谢组学的乌拉尔甘草外泌体特征分析

**DOI:** 10.3724/SP.J.1123.2025.12030

**Published:** 2026-08-08

**Authors:** Yunjun YAO, Xu QIAN, Fuxing SHU, Xichao SONG, Yuanyuan DONG, Leilei JIN, Fengjian LIU, Zuen JI, Jia LIU, Jishuang CHEN

**Affiliations:** 1. 南京工业大学生物与制药工程学院，江苏 南京 211816; 1. College of Biotechnology and Pharmaceutical Engineering，Nanjing Tech University，Nanjing 211816，China; 2. 遵义医科大学生物资源健康利用研究中心，贵州 遵义 563000; 2. Research Center for Healthy Utilization of Biological Resources，Zunyi Medical University，Zunyi 563000，China; 3. 新疆甘草及制品研究重点实验室，新疆 库尔勒 841011; 3. Xinjiang Key Laboratory of Glycyrrhiza Glabra and Products Research，Korla 841011，China; 4. 江苏卫生健康职业学院药学与检验学院，江苏 南京 210029; 4. Department of Pharmacy and Inspection，Jiangsu Health Vocational College，Nanjing 210029，China

**Keywords:** 乌拉尔甘草, 间歇浸没式植物生物反应器, 外泌体, 愈伤组织, 代谢组学, *Glycyrrhiza uralensis*, temporary immersion bioreactor system （TIBS）, exosomes, callus, metabolomics

## Abstract

外泌体是一种纳米级分泌囊泡，其生物活性成分与药效机制研究日益受到重视。乌拉尔甘草（*Glycyrrhiza uralensis*）作为药食同源中药材，富含多种活性成分，但面临资源过度开发与品质不稳定的问题。本研究采用间歇浸没培养技术（TIBS）构建乌拉尔甘草组培体系，并制备其外泌体。以野生甘草茎段为外植体，建立固体培养体系，愈伤组织增殖系数达10.81倍，优化培养基可实现丛生芽再生与无菌苗获得。TIBS体系在5 min/6 h浸没频率下培养28天，愈伤组织增殖系数提升至13.12倍。表征显示，TIBS制备的外泌体呈茶托状，平均粒径56 nm，含量1.07×10^12^ particles/mL，Zeta电位为（-15.030±3.815） mV。采用非靶向代谢组学，在愈伤及其外泌体中共检测到1 760个代谢物，其中差异代谢物1 068个，外泌体中195个代谢物含量高于愈伤组织，主要包括萜类、黄酮类、生物碱、脂肪酸及苯丙酸类等物质。结果表明，TIBS可高效制备均一稳定的乌拉尔甘草外泌体，外泌体活性成分丰富，为后续乌拉尔甘草外泌体在医美、抗炎及抗肿瘤等方向的研究提供材料制备方法与研究基础。

## Abstract (en)

Exosomes are nanometer-sized secretory vesicles that play a pivotal role in mediating intercellular communication. Recently， exosomes derived from herbal medicine have garnered significant attention owing to their unique composition features and therapeutic potential. *Glycyrrhiza uralensis*， a traditional Chinese medicinal and edible plant， is rich in flavonoids， terpenoids， polyketides， and phenolic acids. However， the long-term consequences of overharvesting and artificial cultivation have led to inconsistent quality of licorice materials. Plant tissue culture technology offers a sustainable approach to preserve medicinal plant resources， enabling year-round production independent of seasonal and environmental constraints. To address these challenges and explore the therapeutic potential of herb-derived exosomes， this study employed a laboratory-constructed temporary immersion bioreactor system （TIBS） for *G. uralensis* cultivation and exosomes isolation. The key experimental procedures are summarized as follows： sterile buds derived from *G. uralensis* seeds were used as explants. The callus proliferation medium consisted of Murashige and Skoog medium （MSM） supplemented with 1.18 mg/L 6-benzylaminopurine （6-BA）， 1.44 mg/L naphthaleneacetic acid （NAA） and 1.44 mg/L 2，4-dichlorophenoxyacetic acid （2，4-D）， yielding a maximum proliferation coefficient of 10.81 after 21 days. For shoot proliferation， sterile plantlets were generated on MSM medium supplemented with 1 mg/L 6-BA， 0.05 mg/L NAA， and 150 mL/L coconut milk （CM）， which produced sterile plantlets within 28 days of culture. When cultured in the TIBS system with an immersion frequency of 5 min every 6 h， the callus proliferation coefficient reached 13.12 after 28 days of culture. Exosomes were successfully isolated from the TIBS-cultured *G. uralensis* calli using differential centrifugation and ultracentrifugation protocols. Comprehensive characterization was performed to validate the identity， purity， and stability of isolated exosomes. Morphological observation via transmission electron microscopy （TEM） revealed typical cup-shaped vesicles with a distinct lipid bilayer structure， which is consistent with the canonical exosomes morphology. Dynamic light scattering （DLS） analysis determined an average hydrodynamic diameter of 56 nm. The exosomes preparation exhibited a particle concentration of 1.07×10^12^ particles/mL， and a zeta potential of （-15.030±3.815） mV， indicating excellent colloidal stability suitable for biological applications. To decipher the molecular cargo of these exosomes， untargeted metabolomic profiling was conducted. A total of 1 760 metabolites were identified across callus and exosomes samples， of which 1 068 differential metabolites （DMs） distinguished exosomes from parental calli. Notably， 195 DMs were significantly enriched in exosomes. KEGG （Kyoto Encyclopedia of Genes and Genomes） pathway enrichment analysis revealed enrichment in 11 metabolic pathways， primarily involving terpenoids， flavonoids， alkaloids， fatty acids， and phenylpropanoids. Bioactive compounds of interest included azelaic acid， phloretin， and senkyunolide C， highlighting their potential pharmacological significance. In summary， this study demonstrates that the TIBS-based in vitro platform enables efficient， scalable， and consistent production of *G. uralensis*-derived exosomes with homogeneous morphology and specialized metabolite profiles. The successful isolation， comprehensive characterization， and identification of bioactive metabolite enrichment establish a robust foundation for mechanistic investigations and translational applications. These exosomes exhibited promising therapeutic potential in anti-inflammatory， antioxidant， antitumor， and antibacterial biomedicine. Furthermore， this bioreactor system addresses the quality inconsistency issue in natural *G. uralensis* resources， paving new ways for developing innovative herbal exosome-based nanomedicines.

乌拉尔甘草（*Glycyrrhiza uralensis*）是豆科甘草属植物，具有清热解毒、补脾益气、缓急止痛等功效^［1］^，主要分布于我国西北部地区。甘草酸、甘草素、香豆素、多糖等是甘草中的主要有效成分。目前，野生乌拉尔甘草资源减少，且医药、食品和日化等行业使用广泛、需求量大，难以满足生产需求。组织培养体系的建立为保护乌拉尔甘草资源和进行新品种选育提供了新途径，且有利于提高繁育速度和优良品种的保存。

愈伤组织具有发育成完整植株的能力，因此继代培养愈伤组织并使其发育成再生植株是实现大量繁殖的关键。付丽等^［2］^筛选到了不同外植体诱导愈伤组织的最适培养基；高新新等^［3］^发现下胚轴和子叶是愈伤组织的最佳诱导外植体。间歇浸没式植物生物反应器可将无性繁殖材料在培养液中进行周期性的浸泡培养。这种方式可促进植物组织对营养元素和激素的吸收，提高组培苗的繁殖系数，且人工耗费是固体培养系统的1/2，成本可降低1/5。本实验室自主研发了博方BIO-F系列（bio-function，BIOF）间歇浸没植物生物反应器（temporary immersion bioreactor system，TIBS），并已成功应用于培养栀子^［4］^、半夏^［5］^、石斛^［6］^等名贵中药材。

外泌体（exosomes）是由细胞分泌的纳米级（50~150 nm）双层脂膜囊泡，通过多泡体（multivesicular bodies，MVBs）与质膜融合释放至胞外^［7］^。植物外泌体（plant exosome-like nanoparticles，PENs）的生物发生途径与动物外泌体存在差异，可能通过胞吐作用或质膜直接出芽形成。研究表明，植物外泌体携带植物特异性生物活性分子，包括小RNA（如miR168a）、次生代谢物（酚酸、黄酮类）及防御相关蛋白，可通过跨界调控影响共生微生物或哺乳动物细胞功能。Pomatto等^［8］^从橙汁中提取外泌体作为RNA疫苗的载体，并通过口服和鼻内给药证明了可行性，这为开发新型mRNA疫苗提供了新思路。中草药作为药用植物，含有多种有用的活性成分。目前已有多个关于中药外泌体的研究。例如，灵芝外泌体（ganoderma lucidum-derived exosome-like nanovesicles，GLENVs）经鼻给药可穿透血脑屏障，减轻携带家族性阿尔茨海默病突变的转基因小鼠β淀粉样蛋白沉积与神经炎症，改善认知功能，且无肝肾毒性^［9］^。陈皮中分离出的外泌体（TNVs）可以通过调节肠道菌群和增强肝脂代谢来缓解2型糖尿病诱导的肝脂肪变性^［10］^。机制研究表明，TNVs可以通过增强与脂肪酸氧化相关的基因的表达并抑制与肝脏中脂肪生成和糖酵解相关的基因表达来改善糖脂代谢。机械匀浆是现行植物外泌体制备技术的普遍起始步骤。该操作会破坏细胞结构的完整性，致使大量胞内囊泡与非分泌性组分混杂于提取液中。这些由机械力产生的囊泡群体与通过特定胞泌途径释放的外泌体存在本质区别，但在物理参数上却高度重叠，这为后续的分离纯化带来巨大挑战。此污染问题不仅影响了制品的纯度，更对其后续分子组成与生物学功能的准确解析构成了根本性障碍。使用TIBS技术可以收集纯粹通过胞泌途径释放的外泌体，解决上述问题。并且相较于野外采摘和传统固体培养，TIBS产出的植物材料不受外部气候与琼脂培养基内水分梯度的影响，占地更小且无需大量培养瓶，同时制备的外泌体成分稳定、活性均一。本实验室前期^［11，12］^利用BIO-F系列生物反应器已建立半夏外泌体稳定制备方法，该方法制备的外泌体相较于传统机械匀浆方法制备的外泌体更纯净、粒径更小，具有较高的载药能力和渗透能力。

非靶向代谢组学可系统分析生物样本中检测到的所有代谢产物差异，进而通过标志代谢物的识别推导潜在作用机制^［13］^。近年来，通过高精度和高灵敏度的超高效液相色谱-串联质谱（ultra performance liquid chromatography-tandem mass spectrometry，UPLC-MS/MS）技术，研究人员发现了相关外泌体的应用潜力。Yang等^［14］^通过对马齿苋外泌体（purslane-derived nanovesicles，Po-DENs）进行代谢组学分析发现，Po-DENs含有丰富的亚油酸、佛手酚、银杏酸等多种功能性小分子物质，具有糖尿病皮肤伤口修复的应用潜力，并通过后续细胞实验进行了验证。本研究拟通过TIBS建立乌拉尔甘草组培体系并制备其外泌体，通过基于UPLC-MS/MS的非靶向代谢组学分析构建特征代谢物图谱，筛选差异代谢物并富集代谢通路，解析其空间分布维度的代谢调控网络，为药用资源定向开发提供理论支撑。

## 1 实验部分

### 1.1 仪器、试剂与材料

Vanquish超高效液相色谱仪、Orbitrap Exploris 120质谱仪（美国Thermo Fisher Scientific公司），HT7700透射电子显微镜（transmission electron microscopy，TEM，日本Hitachi公司），A50-Micro纳米流式分析仪（nanoparticle tracking analysis，NTA，英国Apogee公司），Bio-F型间歇浸没植物生物反应器（南京博方生物科技有限公司），E-10D电子天平（赛多斯利科学仪器有限公司），LDZF-30L高压蒸汽灭菌锅（上海申安医疗器械厂），HT-1300-U超净工作台（泰安技术有限公司），移液枪（德国Eppendorf公司），Optima XE-90超高速离心机（美国Beckman Coulter有限公司），PS-60AL超声仪（深圳市雷德邦电子有限公司），JXFSTPRP-24匀浆机（上海净信科技有限公司），LGJ-10C冷冻干燥机（四环福瑞科仪科技发展有限公司）。

次氯酸钠（上海麦克林生化科技股份有限公司）、琼脂粉、蔗糖（国药集团化学试剂有限公司）；萘乙酸（naphthaleneacetic acid，NAA，上海蓝季生物科技有限公司）；2，4-二氯苯氧乙酸（2，4-dichlorophenoxyacetic acid，2，4-D，北京索莱宝科技有限公司）；6-苄氨基嘌呤（6-benzylaminopurine，6-BA，上海伊卡生物技术有限公司）；大量元素培养基（Murashige and Skoog medium，MSM，青岛海博生物科技有限公司）；无水乙醇（分析纯，无锡市亚盛化工有限公司）；甲醇、乙腈、异丙醇、乙酸（色谱纯，上海艾巨酷上海生物科技有限公司）；磷酸盐缓冲液（phosphate buffered saline，PBS，1X，pH=7.4，湖南比克曼控股有限公司）。

将内标配制于混合提取液中用于代谢物的提取，内标的种类与工作浓度如下：吲哚-3-乙-2，2-D_2_酸（indole-3-acetic-2，2-D_2_ acid，2.4 μmol/L）、癸酸-D_19_（decanoic acid-D_19_，0.16 μmol/L）、甘氨胆酸-D_4_（glycocholic acid-D_4_，0.09 μmol/L）、邻苯二甲酸二异丁酯-D_4_（diisobutyl phthalate-3，4，5，6-D_4_，3.5 μmol/L）、［²H_5_］-反式玉米素（*trans*-zeatin-D_5_，0.5 μmol/L）均购自上海甄准生物科技有限公司，原始质量浓度均为100 μg/mL。

愈伤组织诱导培养基（callus induction medium，CI）：MSM+6-BA 1.18 mg/L+NAA 1.44 mg/L+2，4-D 1.44 mg/L；丛生芽诱导培养基（adventitious shoot induction medium，SI）：MSM+6-BA 1 mg/L+NAA 0.05 mg/L+150 mL/L椰乳（coconut milk，CM）。

野生乌拉尔甘草种子于2024年采自新疆地区，经新疆甘草及制品研究重点实验室冀祖恩老师鉴定为乌拉尔甘草。

### 1.2 乌拉尔甘草离体培养体系建立

#### 1.2.1 乌拉尔甘草愈伤组织和丛生苗诱导

用75%无水乙醇浸泡新鲜饱满的乌拉尔甘草种子30 s，无菌水冲洗3次，再用1%次氯酸钠浸泡8 min。随后，用无菌水冲洗3次，每次30 s，无菌滤纸吸干表面水分后接种到蔗糖质量浓度为30 g/L的MS固体培养基上，置于25 ℃恒温组培室中培养，光照强度为2 500 lx、光照时间为14 h/d。

以上述萌发无菌苗为外植体，用无菌解剖刀和镊子将幼茎切成约1 cm长，接种到CI和SI内，CI配方参考付丽等^［2］^的优化结果，SI配方参考葛淑俊等^［15］^和刘惠东等^［16］^的优化结果。每瓶接种4个外植体。将接种后组培瓶置于25 ℃，愈伤组织暗培养箱中培养10天；丛生苗光照培养，光照强度2 500 lx、光照条件14 h/d。

取诱导出的愈伤组织和丛生苗在超净工作台上，用灭过菌的镊子取出，置于事先经高压灭菌干燥冷却后的牛皮纸上，用灭过菌的解剖刀和镊子将愈伤组织切成约1 cm大小的小块，将丛生苗切为约1 cm 长的带芽茎段。分别接种到经121 ℃高压灭菌20 min后的CI和SI培养基中培养，每瓶接种4块愈伤组织或4个带芽茎段，盖上瓶盖后放入恒温组培室内培养，温度为25 ℃，光照强度2 500 lx、光照条件14 h/d。

#### 1.2.2 TIBS培养乌拉尔甘草

取培养21天的上述愈伤组织，在超净工作台上用灭过菌的镊子取出，接种到经121 ℃高压灭菌20 min后无琼脂的培养基内，1 L培养基中接入4瓶愈伤组织，在超净工作台内观察3天，确认无污染后移入TIBS内，设置间歇浸没时间为5 min/6 h。

取培养21天的上述丛生苗，在超净工作台上用灭过菌的镊子取出，置于事先经高压灭菌干燥冷却后的牛皮纸上，用灭过菌的解剖刀和镊子将丛生苗切成约1 cm长的带芽茎段，接种到经121 ℃高压灭菌20 min后无琼脂的培养基内，1 L培养基中接入60个带芽茎段，在超净工作台内观察3天，确认无污染后移入TIBS内，设置间歇浸没时间为5 min/8 h。

### 1.3 乌拉尔甘草外泌体的制备

挑选培养40天长势良好的乌拉尔甘草愈伤组织生物反应器，于超净台中拔去进气口滤头，通过软管倒出液体培养基并分装至50 mL离心管中，将混合物在4 ℃下以300 g离心10 min，以消除组织和细胞碎片。收集上清液，平衡，然后在4 ℃下以5 000 g离心10 min，以进一步去除细胞碎片。将该步骤的上清液转移到新的离心管中，平衡，并在4 ℃下再次以10 000 g离心30 min以去除杂质。最后，将同一样品的上清液放入超速离心管中，平衡，并在4 ℃下以100 000 g离心70 min。除去上清液后，加入1 mL 1×PBS缓冲液，用移液器均匀重悬外泌体，悬浮液储存在-80 ℃长期保存。

### 1.4 乌拉尔甘草外泌体的表征

#### 1.4.1 透射电镜观察外泌体形态

将外泌体取出10 μL，吸取样品10 μL滴加于铜网上沉淀1 min，滤纸吸去浮液。醋酸双氧铀10 μL滴加于铜网上沉淀1 min，滤纸吸去浮液。常温干燥数分钟。在透射电镜中100 kV、5倍镜下进行电镜检测成像。

#### 1.4.2 纳米流式分析仪检测外泌体浓度和粒径

将分离得到的外泌体样本用1×PBS缓冲液适当稀释后进行表征。随后使用纳米颗粒追踪分析技术对外泌体的粒径及浓度进行检测。所有测量均在恒温24 ℃、pH值7.0、电导率15 000.00 μS/cm的受控环境中进行。

### 1.5 代谢物提取

外泌体 向外泌体悬浮液中加250 μL水，涡旋混匀30 s，加入钢珠，放入匀浆仪中匀浆（35 Hz，4 min），重复3次，转入细胞破碎仪中破碎15 min；取50 μL匀浆液用于BCA蛋白定量；取剩余匀浆液200 μL，加入800 μL -40 ℃预冷的提取液（甲醇∶乙腈=1∶1（体积比）），提取液含同位素标记内标，涡旋混匀30 s，超声10 min（冰水浴），-40 ℃静置1 h；样品于4 ℃、12 000 r/min，离心15 min，取上清900 μL于2.0 mL EP管，氮吹，旋干，按比例加入复溶液（甲醇∶乙腈∶水=2∶2∶1（体积比）），样品于4 ℃、12 000 r/min，离心15 min；取上清100 μL于进样小瓶。

愈伤组织 称取样本25 mg，加入1 000 μL -40 ℃预冷的提取液（甲醇∶乙腈∶水=2∶2∶1（体积比）），提取液含同位素标记内标；加入钢珠，放入匀浆仪中匀浆（35 Hz，4 min），再转移到冰水浴超声5 min，此步骤重复3次；-40 ℃静置1 h；样品于4 ℃、12 000 r/min，离心15 min；取上清100 μL于进样小瓶。

### 1.6 分析条件

#### 1.6.1 色谱条件

色谱柱：Phenomenex Kinetex C18色谱柱（50 mm×2.1 mm， 2.6 μm）；柱温：25 ℃；流动相：（A）0.01%乙酸水溶液，（B）异丙醇∶乙腈=1∶1（体积比）；流速：0.3 mL/min；样品室温度：4 ℃。梯度洗脱程序：0~1 min，95%B；1~14 min，95%B~65%B；14~16 min，65%B~40%B；16~18 min，40%B；18~18.1 min，40%B~95%B；18.1~23 min，95%B。进样量：2 μL。

#### 1.6.2 质谱条件

离子源：电喷雾电离（ESI）源，正负离子切换模式；扫描范围：*m/z* 60~1 200；鞘气压力为413.69 kPa，辅助气压力为413.69 kPa，气帘气压力为206.84 kPa，雾化温度为320 ℃；采集方法：信息依赖型采集（information-dependent acquisition，IDA）模式；离子累积时间：50 ms，喷雾电压：正模式3.8 kV，负模式-3.4 kV，碰撞能：40 eV，分辨率15 000。

#### 1.6.3 质量控制

为监测仪器稳定性并校正分析过程中的信号漂移，制备混合所有待测样本的质控（quality control，QC）样本。在样本序列中，每10个待测样本后插入一个QC样本。序列开始时，先连续进样5次QC样本以平衡系统。

#### 1.6.4 数据预处理和搜库

采用Xcalibur（版本：4.4，Thermo）软件提取UPLC-MS/MS质谱峰、积分、保留时间、质荷比和峰强度等，再利用特征峰搜库（http://www.hmdb.ca/）鉴定，根据结构匹配度（相似度）鉴定代谢物（误差小于1×10^-5^）。数据矩阵用80%规则来去除缺失值，即保留至少一组样品中非零值80%以上的变量，再进行空缺值填补（原始矩阵中最小值的一半填补空缺值）。利用内标对质谱峰的响应强度进行归一化，删除QC保留相对标准偏差（RSD）>30%的变量，数据进行以10为底的对数化处理，得到的数据矩阵用于后续分析。

#### 1.6.5 差异代谢物及代谢通路分析

原始数据经ProteoWizard软件转成mzXML格式后，使用北京擎科公司合作编写的R包进行无监督的主成分分析法（principal componebt analysis，PCA）、正交偏最小二乘法（orthogonal partial least squares discrimination analysis，OPLS-DA，置换校验次数为200）、Student’s *t*检验和差异倍数分析。差异代谢物的选择基于OPLS-DA模型得到的变量权重值（variable importance in projection，VIP）和Student’s *t*检验*p*值来确定，VIP>1.0、*p* <0.05，倍数变化值（FC）≥2或≤0.5的代谢物为差异代谢物。通过京都基因与基因组百科全书（Kyoto Encyclopedia of Genes and Genomes，KEGG）数据库（https://www.kegg.jp/kegg/pathway.html）进行差异代谢物的代谢通路注释，Python软件包Scipy.stats进行通路富集分析。

### 1.7 乌拉尔甘草生长指标的测定

取固体培养和TIBS中的愈伤组织和组培苗，愈伤组织在电子天平上称取培养前后的鲜重比较增殖系数；组培苗用直尺测量株高、节间距，并记录茎节数作为甘草的生长指标。

## 2 结果与讨论

### 2.1 乌拉尔甘草组培体系的建立

以乌拉尔甘草种子萌发获得的幼苗茎段为外植体，进行愈伤和丛生芽诱导（图1）。在愈伤诱导培养基中，经10天暗培养和11天光照培养可获得疏松愈伤，乌拉尔甘草愈伤外观形态呈现淡黄颜色，质地致密。将生长状态良好的愈伤组织转入TIBS中培养，培养40天，增殖系数达13.12，是组培瓶的1.21倍。带芽茎段诱导出的丛生芽，分别在固体瓶和TIBS中培养，幼苗的平均株高分别为（4.65±0.94） cm和（16.79±2.60） cm。同时培养过程中，观察到利用TIBS培养愈伤组织和丛生芽可延长褐化发生时间。组培瓶培养时，愈伤组织和丛生芽分别在21~23天和26~28天开始褐化，而在TIBS培养时可以延长到38~40天和58~60天才逐渐褐化。

**图1:**
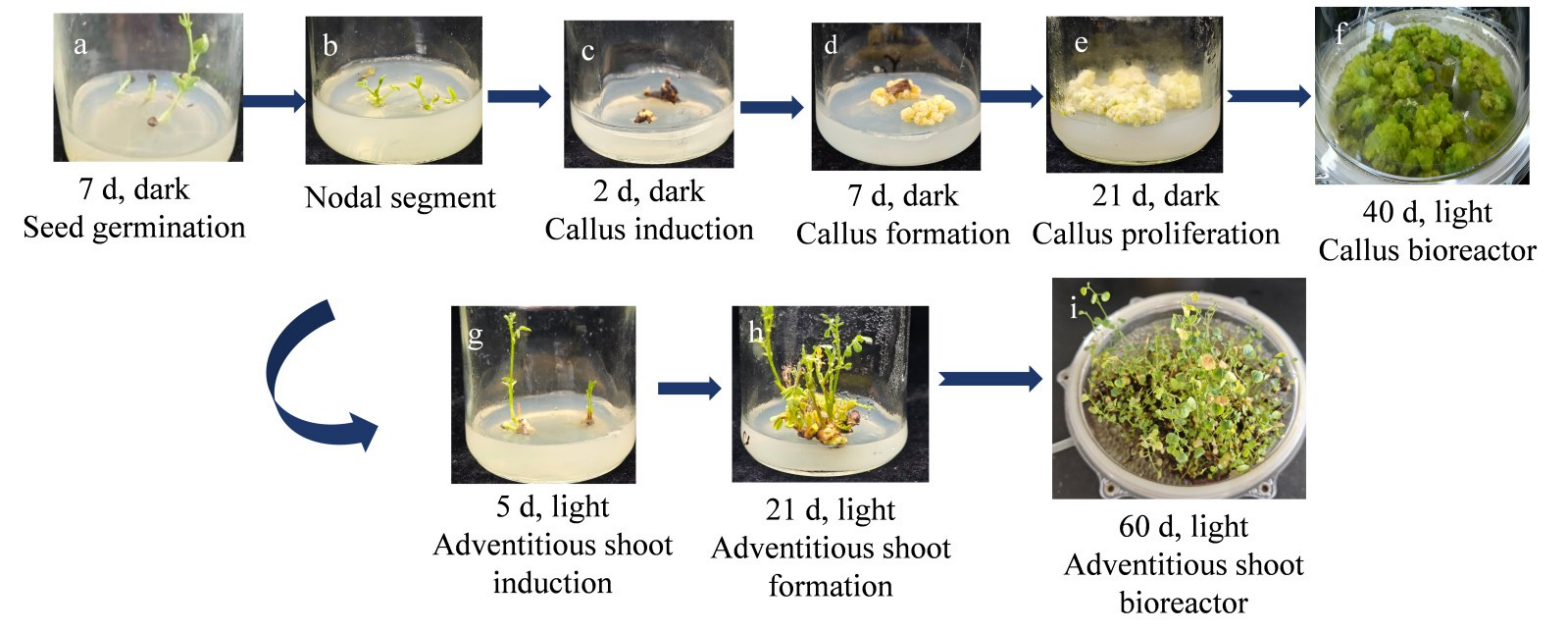
（a~f）乌拉尔甘草愈伤和（a，b，g～i）丛生芽的培养流程图

对上述固体和间歇浸没培养丛生苗的株高、节间距及茎节数进行统计学分析（图2）。在TIBS中培养的丛生芽株高和节间距为（16.79±2.60） cm和（1.93±0.61） cm，相较于固体瓶中分别提高了260%和141%（*p*<0.05）。而这两种培养方式下，组培瓶中茎节数（平均为3.14个）略高间歇浸没培养下茎节数（平均为2.88个）。这种差异主要在于，组培瓶相较于TIBS空间更为狭小，植物产生的乙烯等气体容易在瓶内积累，形成诱导外植体分化、丛生芽增殖的理想条件^［17］^。因而，组培瓶中甘草茎节数略高于反应器培养。TIBS系统空间较大，更利于幼苗的培养。故而，其丛生芽株高和节间距高于固体瓶培养。由此可见，利用TIBS间歇浸没培养系统对提高乌拉尔甘草鲜重、株高、节间距和增殖系数具有良好促进作用，可为大规模短时间获取乌拉尔甘草组培幼苗提供可能。

**图2:**
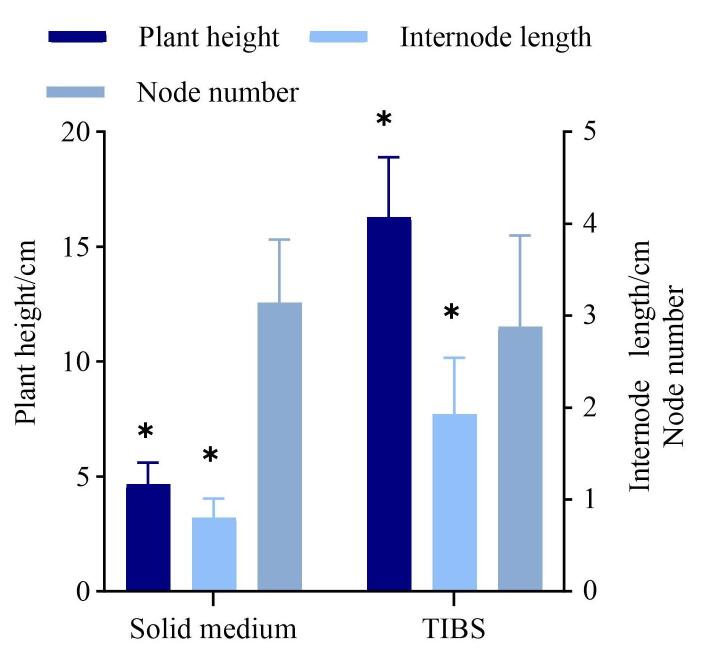
两种培养条件下乌拉尔甘草丛生芽的株高、节间距及茎节数的柱状图（*n*=6）

### 2.2 乌拉尔甘草外泌体的表征分析

对TIBS制备获得的乌拉尔甘草进行形貌、粒径分布、Zeta电位和颗粒浓度表征分析。由图3a观察可得：TIBS制备的乌拉尔甘草外泌体在透射电镜下其外观形态中间呈凹陷的茶托状小囊泡，形态均一，脂质双层结构清晰。NTA结果显示：TIBS制备的乌拉尔甘草外泌体集中分布在50～90 nm（图3b），中位粒径为56 nm，最大粒径为200 nm，符合典型外泌体粒径特征，D90/D10值为1.4，接近于1，反映TIBS制备的乌拉尔甘草外泌体尺寸高度均一。该粒径与Xu等^［18］^报道的乌拉尔甘草外泌体粒径范围（153.47±3.29） nm相比，具有一定差异。TIBS制备的外泌体粒径低于该范围，这可能与提取方法及组织来源特异性有关：外泌体来源于野生乌拉尔甘草的根部，采用研磨破碎法进行提取，该过程中由于外部剪切力的作用可能导致细胞结构破坏，进而释放出部分较大的细胞内囊泡。相比之下，本研究中的外泌体则通过收集TIBS内愈伤组织的培养液获得，从而有效避免了机械剪切力的干扰，确保所获得的外泌体完全依赖于细胞的胞吐作用释放至胞外。外泌体总颗粒浓度为1.07×10^12^ particles/mL，并测得Zeta电位为（-15.030±3.815） mV（图3c），符合植物外泌体典型表面电荷特征（-10~-50 mV）^［8，11］^，Zeta电位反映了外泌体表面净电荷和颗粒稳定性^［19］^，在-10~-30 mV 范围内体系相对稳定，适于短期储存。

**图3:**
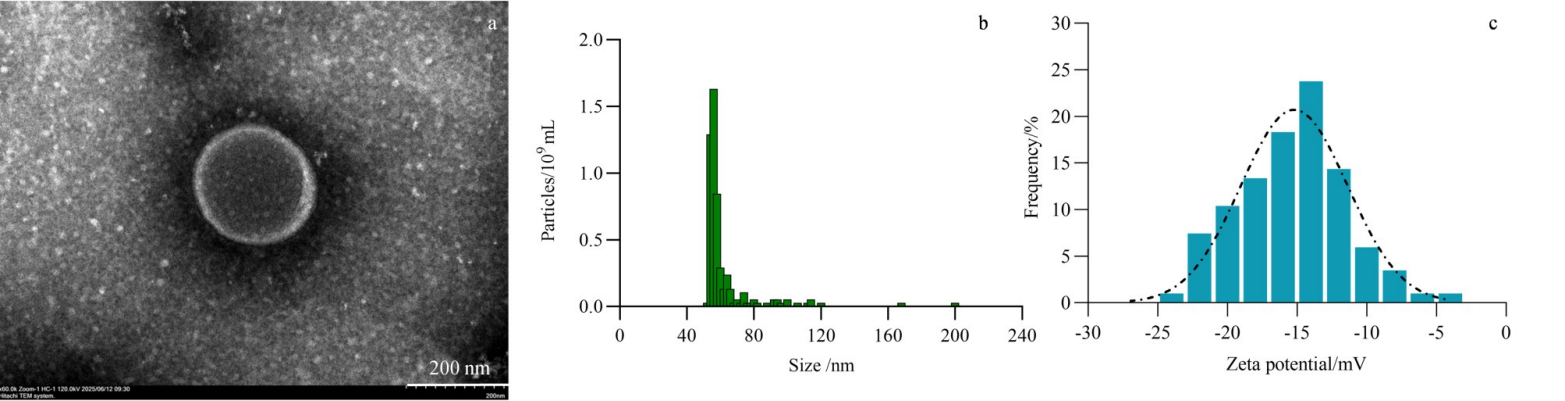
外泌体的（a）透射电镜图、（b）粒径分布图和（c）Zeta电位图

### 2.3 外泌体代谢产物谱

采用UPLC-MS/MS对乌拉尔甘草愈伤组织和外泌体进行检测。在正、负离子模式下，QC样品的总离子流图（TIC）见图4。结果表明，目标分析物在设定的色谱条件下实现了良好的色谱分离，色谱峰形对称，峰形尖锐且无明显拖尾现象，基线平稳无杂峰干扰，各有效色谱峰分离度佳，响应信号强度适宜，表明色谱分离条件与质谱检测参数优化合理，可满足后续代谢物定性、定量分析的技术要求，为样品中化学成分的精准表征提供了可靠的数据支撑。研究植物组织及其外泌体代谢产物的差异，可为开发外泌体载药提供研究基础。愈伤组织和外泌体中共检测出1 760个化合物。这些化合物在HMDB数据库中可细分为萜类、聚酮、莽草酸及苯丙酸类、生物碱、碳水化合物、氨基酸及短肽化学物，按类别可区分为四级化合物（218种）、三级化合物（56种）和二级化合物（8种）。采用PCA和OPLS-DA对愈伤和外泌体中代谢物进行差异分析，从而观察愈伤组织和外泌体代谢物的自然聚类趋势。PCA结果（图5a）显示，甘草愈伤组织的成分和培养液外泌体的成分在维度上有一定的分离趋势。模型评价参数*R* ^2^ *Y*、*Q* ^2^分别为0.999、0.996，均远大于0.5，表明模型稳定且可靠性极高（图5b）。该结果证实OPLS-DA模型构建成功，甘草愈伤组织与甘草愈伤来源外泌体样品在得分图中呈现清晰且良好的分离趋势，二者的代谢组学特征存在显著差异。

**图4:**
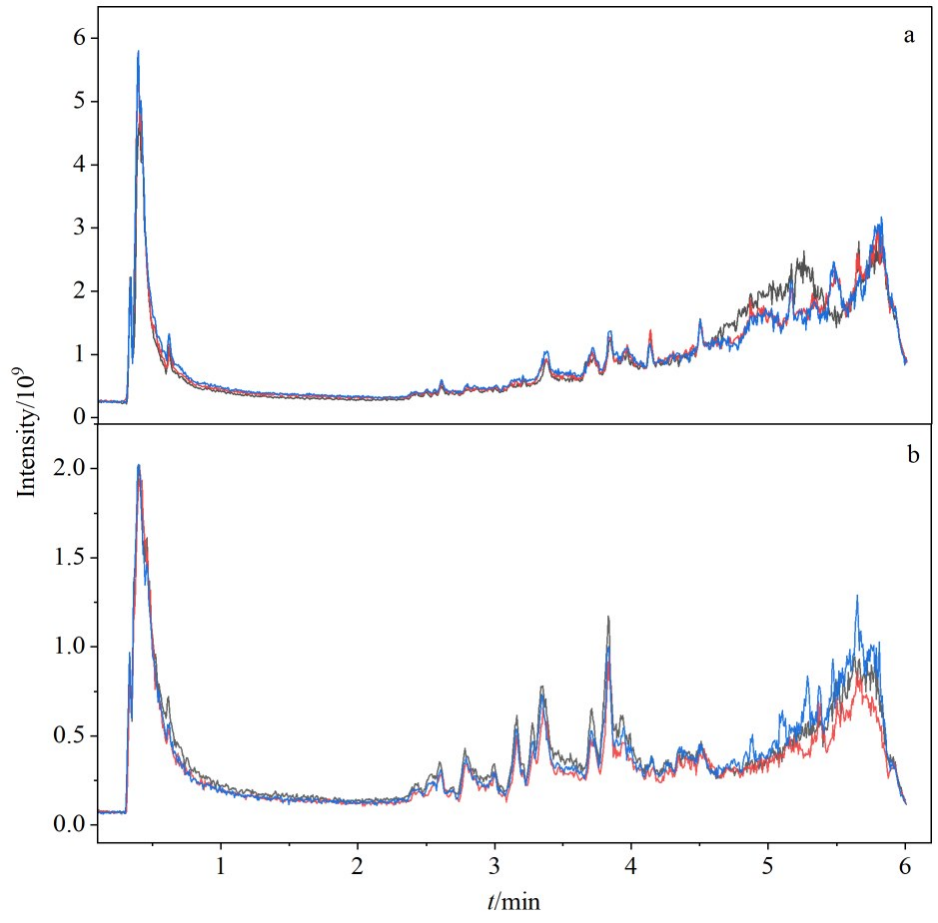
在（a）正、（b）负离子模式下QC样本的TIC图

**图5:**
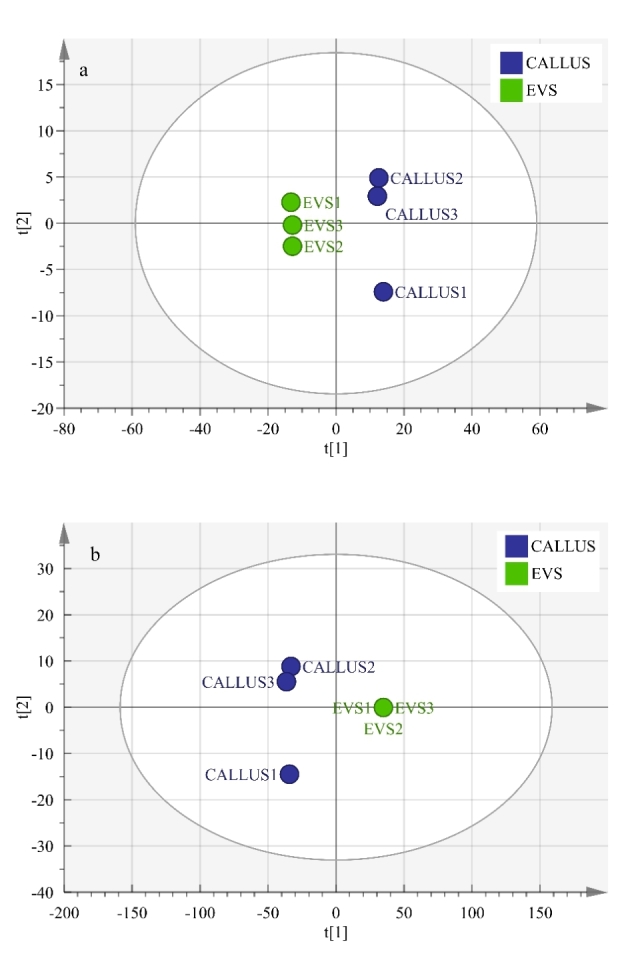
两样本间的代谢物（a）PCA和（b）OPLS-DA评分图

#### 2.3.1 差异代谢物筛选

为进一步了解乌拉尔甘草愈伤组织和外泌体中的代谢物变化，以VIP值>1、FC≥2或≤0.5，且*p*值<0.05为阈值筛选差异代谢物，获得差异代谢物信息绘制火山图。由图6差异代谢物火山图可知，与愈伤组织相比，外泌体中有1 068个差异代谢物。其中，195个代谢物上调，主要包括壬二酸、根皮素、穿心莲内酯、愈伤酸、洋川芎内酯C、植烷酸、咖啡酸等有效活性成分；873个代谢物下调，主要包括橙皮苷、水杨酸、甘草素、圣草酚、芹菜苷、甘草黄酮C等有效活性成分。结果表明，差异代谢物主要富集在愈伤组织内，少量差异代谢物随外泌体分泌到胞外（*p*<0.05）。咖啡酸属于苯丙烷类化合物，在植物系统中广泛参与抗氧化防御和次生代谢途径。其在外泌体中的上调（FC=9.36）可能表明外泌体在介导细胞间信号传递时，增强了抗氧化应激或抗炎反应。咖啡酸可通过抑制活性氧积累和调节相关酶（如过氧化物酶）的活性，发挥保护作用，这可能与外泌体在应激响应中的功能相关，例如在植物发育或损伤修复过程中，外泌体可能通过富集此类代谢物来促进细胞存活和组织再生。

**图6:**
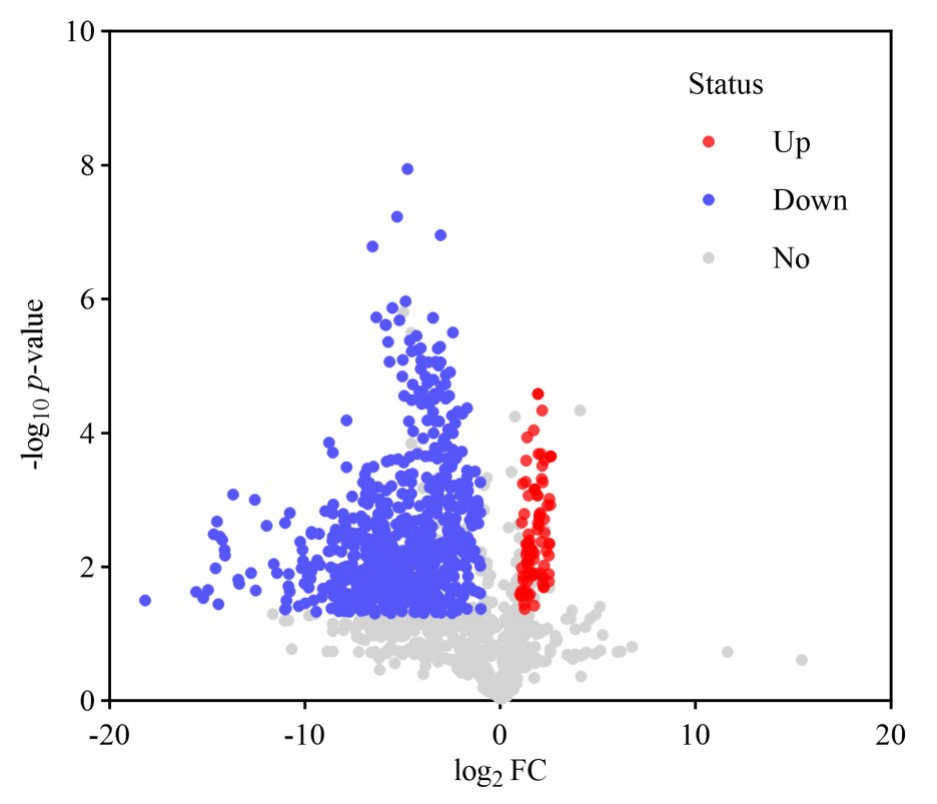
乌拉尔甘草愈伤组织和外泌体差异代谢物火山图

#### 2.3.2 乌拉尔甘草愈伤外泌体中代谢物代谢通路

进一步对乌拉尔甘草愈伤组织外泌体中优势代谢物进行KEGG富集分析，结果如图7a所示，代谢物显著富集在类黄酮生物合成、ABC转运蛋白、氨基酸生物合成、乙醛酸盐和二羧酸盐代谢、D-氨基酸代谢、亚油酸代谢、不饱和脂肪酸生物代谢、苯丙烷类生物合成等11条代谢通路。其中最显著的就是类黄酮生物合成、ABC转运蛋白、氨基酸生物合成这3条代谢通路。较多代谢物参与类黄酮生物合成，其中包括甘草素、短叶松素、异甘草素、柚皮素7-*O-β*-D-葡萄糖苷、根皮素、山柰酚、圣草酚、花旗松素等15种活性物质。类黄酮生物合成通路的激活通常与抗氧化防御、抗逆胁迫及生物互作密切相关，其富集可能反映机体通过合成黄酮类次级代谢产物（如根皮素、圣草酚）抵御氧化损伤或病原侵袭，同时暗示苯丙氨酸代谢流的重新分配。

**图7:**
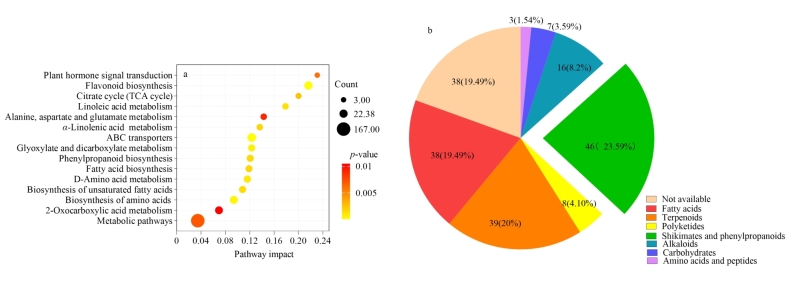
外泌体优势代谢物的（a）KEGG富集通路图和（b）分类饼图

#### 2.3.3 乌拉尔甘草外泌体有效活性物质分析

使用 HMDB 数据库的化合物分类功能，对乌拉尔甘草愈伤组织外泌体的优势差异代谢物进行分类。由优势差异代谢物化合物分类图可知（图7b），38个脂肪酸占19.4%，38个萜类占19.4%，8个聚酮占4.1%，46个莽草酸及苯丙酸类占23.59%，16个生物碱占8.20%，7个碳水化合物占3.59%，3个氨基酸及短肽占1.54%。可以发现，外泌体中最多的代谢物类别为莽草酸及苯丙酸类代谢物，排名第二的为脂肪酸，推测原因为脂肪酸易分泌到胞外且易溶于水。莽草酸及苯丙酸类是植物体内主要的抗氧化^［20］^、抗炎、抗菌与信号分子，外泌体富集莽草酸及苯丙酸类代谢物，说明其主要功能是将这些活性分子精准递送至靶细胞，这揭示了甘草愈伤组织外泌体主要参与次生代谢调控、逆境防御响应与胞间信号传递，同时提示其可能承载甘草愈伤组织抗氧化、抗炎等核心药用活性物质的转运与递送功能。

乌拉尔甘草愈伤组织与外泌体里含有多种具有美白抗炎作用的活性物质，包括橙皮苷、橙皮素单葡萄糖苷（HMG）、甘草查耳酮A、甘草素、芹菜苷等，其中大部分是黄酮类物质（16种），还有4种萜类、1种环状聚酮类、1种酚酸类、1种脂肪酸及衍生物类。可以看出，活性成分主要富集在愈伤组织内（见图8和表1）。如橙皮苷，作为疏水性黄酮类化合物，其低效跨膜扩散特性导致其在外泌体中仅微量检出，log_2 _FC值达到了-8.43，表明该类物质主要定位于细胞内膜系统或液泡中发挥细胞内抗氧化功能。橙皮苷有抗炎抗氧化^［21］^等多种功效，抗氧化性体现在可以抑制机体内过多的自由基，橙皮苷还可以通过对免疫系统的调节实现抵抗炎症的作用。HMG与橙皮苷结构相似、作用类似，并且水溶性与胃肠道吸收度都远高于橙皮苷^［22］^，除此之外，HMG还具有较强的抗感冒病毒活性。壬二酸作为一种小分子有机酸，其在外泌体中的显著富集（*p*<0.05，log_2 _FC=1.29）可能依赖于ABC转运体介导的主动包装机制，提示外泌体通过选择性分泌该分子调控培养液微环境的抗菌活性及pH稳态。甘草素、甘草查耳酮A和甘草次酸是乌拉尔甘草的特征性活性成分，其中甘草素和甘草查耳酮A^［23］^已被证明可以通过多种机制对不同的癌细胞有抗肿瘤作用。除了上述两种甘草黄酮类物质，甘草次酸^［24］^也被国内外大量研究证明具有如抗炎、抗氧化、神经保护等多种生物活性。芹菜苷、山柰酚、根皮素等均是广泛存在于蔬菜、水果和中草药中的天然抗氧化剂，其中根皮素在外泌体中有更多的积累。除了抗氧化外，根皮素^［25］^还具有杀菌、消炎、抗肿瘤、降血糖、抑制酪氨酸酶等多种活性。水杨酸^［26］^、壬二酸^［27］^均作为美白祛痘成分广泛应用于护肤品中，壬二酸水溶性和配伍性较差，这限制了它在化妆品方面的应用，外泌体作为特殊的脂质小分子，也许能成为一种壬二酸的新型作用方式。

**图8:**
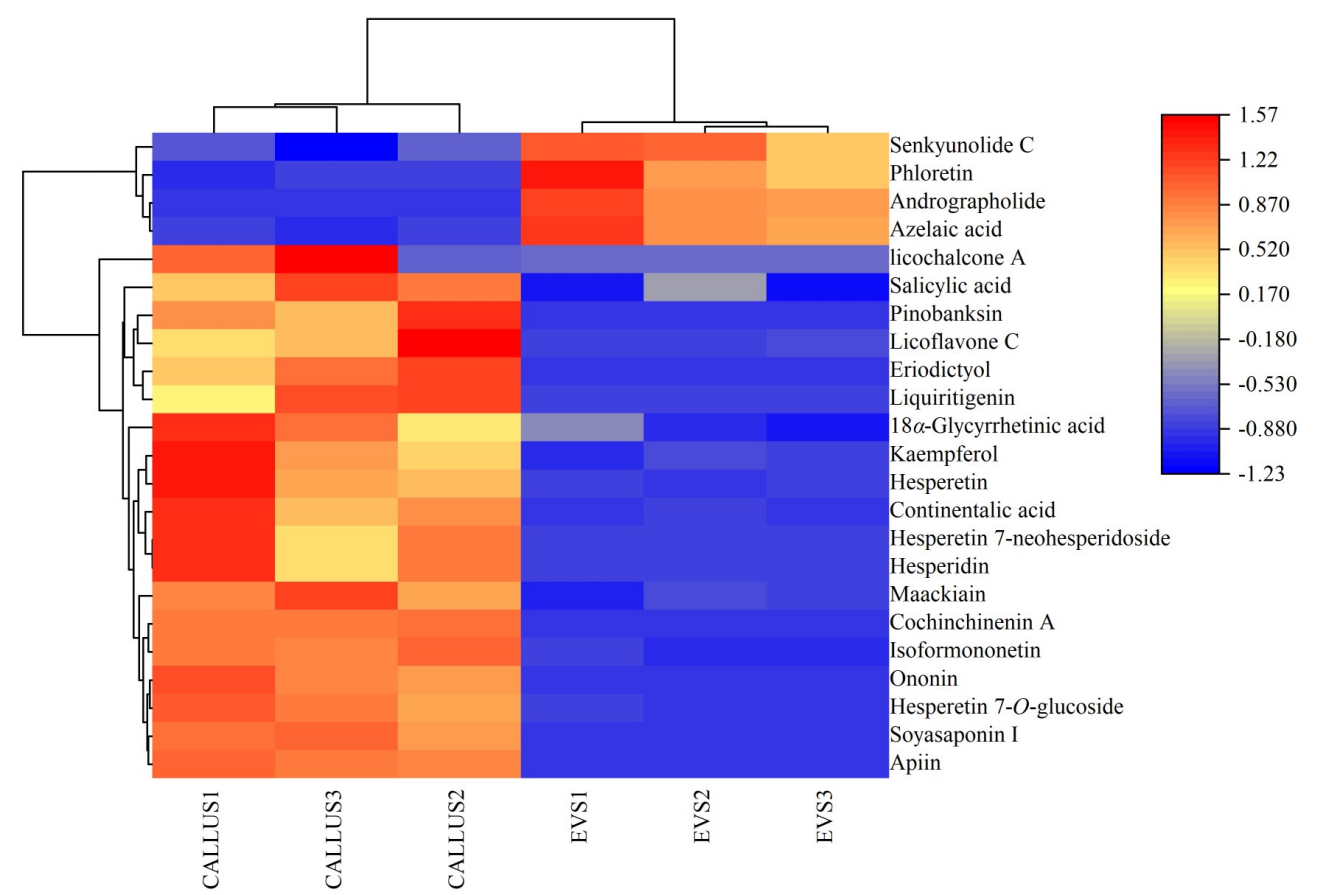
差异代谢物热图

**表1:** 乌拉尔甘草愈伤组织与外泌体主要有效活性成分

Metabolite	*m/z*	*t*_R_/s	Chemical formula	VIP	*p*-Value	log_2_ FC	*M*_r_
Hesperidin （橙皮苷）	611.196	180.900	C_28_H_34_O_15_	1.165	0.023	-8.429	610.562
Licochalcone A （甘草查耳酮A）	337.144	246.400	C_21_H_22_O_4_	0.142	0.196	-5.787	314.385
Liquiritigenin （甘草素）	255.066	215.000	C_15_H_12_O_4_	1.175	0.029	-4.033	244.252
Cochinchinenin A （剑叶龙血素A）	285.113	222.600	C_17_H_18_O_4_	1.174	0.000	-8.570	286.322
Hesperetin 7-*O-*glucoside （橙皮素单葡萄糖苷）	463.124	205.700	C_22_H_24_O_11_	1.179	0.004	-4.386	464.409
Phloretin （根皮素）	257.080	196.000	C_15_H_14_O_5_	1.152	0.023	0.873	274.268
Eriodictyol （圣草酚）	269.046	217.100	C_15_H_12_O_6_	1.184	0.013	-5.710	288.257
Apiin （芹菜苷）	545.130	196.300	C_26_H_28_O_14_	1.186	0.001	-5.560	388.367
Ononin （芒柄花苷）	431.133	192.000	C_22_H_22_O_9_	1.185	0.003	-7.073	430.404
Licoflavone C （甘草黄酮C）	339.122	248.000	C_20_H_18_O_5_	1.033	0.045	-7.106	338.353
Pinobanksin （短叶松素）	271.061	235.900	C_15_H_12_O_5_	1.185	0.014	-7.026	272.251
Maackiain （高丽槐素）	285.075	224.300	C_16_H_12_O_5_	1.104	0.001	-3.354	284.264
Neohesperidoside （新橙皮苷）	611.196	180.900	C_28_H_34_O_15_	1.165	0.023	-8.429	302.279
Hesperetin （橙皮素）	301.072	231.900	C_16_H_14_O_6_	1.179	0.022	-4.829	286.236
Kaempferol （山柰酚）	285.041	187.100	C_15_H_10_O_6_	1.149	0.005	-2.759	204.222
Senkyunolide C （洋川芎内酯C）	203.071	234.400	C_12_H_12_O_3_	1.058	0.002	1.931	188.221
Azelaic acid （壬二酸）	187.097	196.400	C_9_H_16_O_4_	1.174	0.001	1.297	138.121
Salicylic acid （水杨酸）	137.024	175.200	C_7_H_6_O_3_	1.048	0.007	-1.723	943.123
Soyasaponin Ⅰ （大豆皂苷Ⅰ）	941.511	249.000	C_48_H_78_O_18_	1.185	0.002	-4.385	302.451
Continentalic acid （长白楤木酸）	301.217	307.100	C_20_H_30_O_2_	1.173	0.015	-4.347	470.684
18*α*-Glycyrrhetinic acid （甘草次酸）	471.344	241.700	C_30_H_46_O_4_	1.102	0.009	-0.684	350.449
Andrographolide （穿心莲内酯）	351.213	203.000	C_20_H_30_O_5_	1.180	0.007	4.767	610.562

VIP： variable importance in projection.

## 3 结论

本研究利用TIBS体系成功建立了乌拉尔甘草的组织培养系统，并在此基础上制备了源自乌拉尔甘草愈伤组织的外泌体。外泌体的功能活性易受培养和提取方式的影响。TIBS培养体系模拟了植物自然生长环境，其所获外泌体相较于传统机械提取法，粒径偏小具有更高的生物相容性与治疗潜能，值得后续进行药物递送方面的深入研究。我们进一步采用基于UPLC-MS/MS的非靶向代谢组学方法，对愈伤组织及其外泌体进行了代谢物谱分析。结果表明，乌拉尔甘草愈伤组织外泌体具有多种活性成分，部分成分在美白、抗肿瘤、抗炎、抗菌及降血糖等方面将展现出潜在的应用价值。尽管本研究对愈伤组织外泌体进行了系统的表征与代谢物分析，但尚未开展关于其功能应用层面的实验验证。后续研究中，我们希望利用小鼠黑色素瘤细胞模型（B16F10）检测外泌体干预前后酪氨酸酶活性、黑色素含量及相关信号通路关键蛋白的表达，系统评估甘草外泌体的美白功效及机制；同时，也借助斑马鱼模型开展体内安全性评价与活体成像观察，全面分析其生物相容性及组织分布特征。在此基础上，细胞摄取实验、示踪技术和药物装载实验也会被后续应用于探究该外泌体作为天然药物递送载体的潜力，为开发基于乌拉尔甘草愈伤组织外泌体的功能性产品提供理论依据。
